# Association Between End-Tidal Carbon Dioxide and Pulmonary Artery Pulse Pressure During Veno-Arterial Extracorporeal Membrane Oxygenation with Impella^®^: A Retrospective Exploratory Study

**DOI:** 10.3390/jcm15208070

**Published:** 2026-10-17

**Authors:** Hayato Shimizu, Chikara Ishino, Tomoya Ota, Naohiro Seki, Shunsuke Suzuki, Eriko Gojo, Kohei Sakagami, Miho Nagai, Junji Kakizaki, Yuta Hirano, Ken Magata, Goji Shimizu, Manato Watanabe, Yusuke Kuwahara, Chihiro Kagohashi

**Affiliations:** 1 Department of Clinical Engineering, Japanese Red Cross Musashino Hospital, Kyonan-cho 1-26-1, Musashino City 180-8610, Tokyo, Japan; 2 Emergency and Critical Care Center, Japanese Red Cross Musashino Hospital, Kyonan-cho 1-26-1, Musashino City 180-8610, Tokyo, Japan; 3 Intensive Care Unit, Japanese Red Cross Musashino Hospital, Kyonan-cho 1-26-1, Musashino City 180-8610, Tokyo, Japan; 4 Department of Thoracic Surgery, Japanese Red Cross Musashino Hospital, Kyonan-cho 1-26-1, Musashino City 180-8610, Tokyo, Japan

**Keywords:** V-A ECMO, Impella, pulmonary artery catheter, pulmonary artery pulse pressure, end-tidal carbon dioxide

## Abstract

**Background:** Assessment of native cardiopulmonary circulation is essential during combined veno-arterial extracorporeal membrane oxygenation (V-A ECMO) and Impella^®^ support (ECPELLA). Pulmonary artery catheter (PAC)-derived pulmonary artery pulse pressure (PAPP) is associated with RV stroke volume and pulmonary arterial compliance; however, PAC monitoring has procedure-related risks and technical limitations. End-tidal carbon dioxide (EtCO_2_) is a simple, continuous, noninvasive indicator of pulmonary blood flow; however, its relationship with PAPP during ECPELLA support remains unclear. **Methods:** We retrospectively analyzed adult patients who underwent simultaneous ECPELLA and PAC monitoring between April 2024 and December 2025. Hemodynamic variables and EtCO_2_ were evaluated on ECPELLA support day 1 and on the final day of V-A ECMO support. Correlations between EtCO_2_ and PAPP, as well as their temporal changes during ECPELLA support, were assessed using Spearman correlation analysis. **Results:** Sixteen patients were included. EtCO_2_ showed significant positive correlations with PAPP on ECPELLA support day 1 (*r* = 0.63, *p* = 0.009), on the final day of V-A ECMO support (*r* = 0.67, *p* = 0.007), and for changes over time (ΔPAPP vs. ΔEtCO_2_, *r* = 0.76, *p* = 0.001). **Conclusions:** EtCO_2_ was associated with PAPP and its temporal changes during ECPELLA support. Although these findings suggest a potential role for EtCO_2_ in noninvasive assessment of PAPP trends, further studies in larger cohorts are needed to better characterize this relationship.

## 1. Introduction

Veno-arterial extracorporeal membrane oxygenation (V-A ECMO) is one of the most widely used forms of temporary mechanical circulatory support (t-MCS) for patients with severe, drug-refractory cardiogenic shock or cardiac arrest, as recommended in recent international guidelines [1,2,3,4,5]. After cardiac arrest, severe and potentially reversible myocardial dysfunction frequently necessitates prolonged dependence on retrograde V-A ECMO flow support [6]. This non-physiological retrograde flow substantially reduces antegrade pulmonary blood flow and increases left ventricular (LV) afterload, resulting in LV overload and pulmonary congestion [7]. To mitigate these unfavorable hemodynamic effects, adjunctive LV venting or unloading using percutaneous microaxial flow pumps such as Impella^®^ is being used more often [8,9]. During V-A ECMO-assisted Impella support (ECPELLA), accurate assessment of right ventricular (RV) function is essential, since adequate RV performance is required to maintain antegrade pulmonary flow, facilitate effective Impella operation, and enable safe weaning from V-A ECMO [1,2,10].

Pulmonary artery catheter (PAC) monitoring remains the clinical standard for continuous evaluation of pulmonary circulation and RV performance, providing parameters such as mixed venous oxygen saturation (SvO_2_), native cardiac output (Native-CO), and pulmonary artery pressure (PAP) [1,2,3,4,11]. Despite its utility, PAC monitoring has several limitations, such as infection, catheter kinking, thrombosis, and measurement errors [11,12]. Noninvasive alternatives, including central venous oxygen saturation (ScvO_2_) and echocardiographic estimation of Native-CO and PAP, require intermittent measurement and advanced technical skill [12,13,14,15,16]. These limitations are particularly pronounced during t-MCS support [17,18].

End-tidal carbon dioxide (EtCO_2_) has emerged as a continuously obtainable, noninvasive parameter that reflects pulmonary blood flow and ventilation–perfusion matching. Resuscitation guidelines suggest that higher EtCO_2_ values (>20–25 mmHg) during cardiopulmonary resuscitation (CPR) reflect improved perfusion and a greater likelihood of return of spontaneous circulation (ROSC) [19,20]. Several studies of cardiopulmonary bypass (CPB) and V-A ECMO settings have also reported correlations between EtCO_2_ and Native-CO, radial pulse pressure (rPP), and pulmonary blood flow [17,18,21]. However, the accuracy of thermodilution-based Native-CO measurement decreases under unstable hemodynamic conditions [22,23]. Likewise, rPP is attenuated, limiting its reliability as a marker of native cardiac function during Impella support.

Under the limitations imposed by ECPELLA support, PAP, a pressure-based index, can be used as a marker of native cardiac function, particularly for assessing recovery of RV function from ECPELLA initiation through V-A ECMO weaning [10]. Pulmonary artery pulse pressure (PAPP), derived from the pulse pressure of the pulmonary artery, reflects RV stroke volume and pulmonary arterial compliance [24]. This parameter has been reported, including in the ELSO registry, as a prognostic marker in V-A ECMO populations [25]. However, the relationship between EtCO_2_ and PAPP under ECPELLA support has not been fully elucidated. This study aimed to evaluate the association between EtCO_2_ and PAPP during ECPELLA support.

## 2. Materials and Methods

### 2.1. Study Design and Population

This retrospective observational study included adult patients (≥18 years) who underwent simultaneous V-A ECMO, Impella CP, and PAC insertion at the Japanese Red Cross Musashino Hospital between April 2024 and December 2025. Patients receiving V-A ECMO for both extracorporeal cardiopulmonary resuscitation (ECPR) and non-ECPR indications were included. Patients supported with veno-venous ECMO (V-V ECMO), veno-arterial-venous ECMO (V-AV ECMO), or Impella 5.5 were excluded.

The study was conducted in accordance with the Declaration of Helsinki, and approved by the Institutional Review Board of Japanese Red Cross Musashino Hospital (protocol code 7048; date of approval: 11 December 2025). The requirement for informed consent was waived because of the retrospective design. Information regarding this study was made publicly available on the hospital website to allow patients to opt out.

### 2.2. Hemodynamic Measurements

Demographic characteristics, clinical background, and hemodynamic variables were retrospectively collected from the electronic medical records. Only time points at which both PAC-derived parameters and EtCO_2_ were simultaneously recorded were eligible for analysis. Hemodynamic data were extracted at two predefined time points. ECPELLA support day 1 was defined as the first available simultaneous measurement of PAC-derived parameters and EtCO_2_ after ECPELLA initiation and admission to the intensive care unit. The final day of V-A ECMO support was defined as the last available simultaneous measurement of these parameters while the patient remained on V-A ECMO, regardless of whether the patient subsequently underwent V-A ECMO decannulation. Changes in hemodynamic and respiratory variables between these two time points (Δ values) were evaluated to assess temporal changes during V-A ECMO support.

Hemodynamic measurements included PAP (systolic, diastolic, and mean), right atrial pressure (RAP; if RAP was unavailable, central venous pressure [CVP] was used as a surrogate), and Native-CO obtained by thermodilution. The pressure transducer was leveled and zeroed twice daily and readjusted as necessary after changes in patient position or patient transport. The PAP values displayed on the bedside monitor and recorded in the electronic medical record were calculated by the monitor as averages over eight cardiac beats. PAPP was calculated as follows:

*PAPP* = *Systolic PAP* − *Diastolic PAP*, (1)

All patients were mechanically ventilated using a pressure-controlled assist/control mode on the HAMILTON-C6 ventilator (Hamilton Medical AG, Bonaduz, Switzerland). Ventilation was adjusted to maintain a target arterial carbon dioxide tension (PaCO_2_) of 35–45 mmHg. Inspiratory pressure and respiratory rate were modified as needed, while ECMO sweep gas flow was titrated in parallel to achieve the PaCO_2_ target.

Arterial blood gas analyses, including PaCO_2_ measurements, were obtained from right radial artery samples as part of routine clinical care. Because arterial blood gas sampling and hemodynamic monitoring were not performed simultaneously, the closest arterial blood gas measurement obtained during ongoing ECPELLA support within a 2-h window was matched to each PAP and EtCO_2_ measurement. The 2-h window was selected to reflect the routine monitoring intervals at our institution. EtCO_2_ was measured using a mainstream capnography sensor integrated into the ventilator circuit, which was calibrated before each use. The displayed EtCO_2_ value was automatically calculated by the ventilator for each respiratory cycle.

Data with missing PAP or EtCO_2_ values, evident measurement artifacts, or physiologically implausible readings were excluded from the analysis.

### 2.3. V-A ECMO and Impella Management

V-A ECMO support was provided using the SP-200 system (Terumo Corporation, Tokyo, Japan). V-A ECMO was established using a 21 Fr drainage cannula inserted via the femoral vein and a 16.5 Fr arterial return cannula via the femoral artery. All patients were supported with an Impella CP device (Abiomed, Inc., Danvers, MA, USA). The total flow of the combined ECPELLA system was titrated to maintain adequate systemic perfusion, targeting a mean arterial blood pressure (MAP) of >65 mmHg, improving lactate levels, and increasing SvO_2_, rather than adhering to fixed ECMO blood flow (ECBF) settings. During severe and potentially reversible myocardial dysfunction, ECBF was maximized while maintaining a pump head pressure <400 mmHg to achieve total LV unloading. Once PAPP improved, ECBF was gradually reduced while increasing antegrade Impella pump flow. The Impella was maintained at the highest possible setting without inducing suction events, and low-dose dobutamine was occasionally administered during V-A ECMO weaning to facilitate transition to Impella-only support.

### 2.4. Statistical Analysis

Continuous variables are presented as medians (interquartile range), and categorical variables as counts and percentages. Comparisons between patients with and without PAC placement were performed using the Mann–Whitney U test for continuous variables and Fisher’s exact test or the Fisher–Freeman–Halton exact test, as appropriate, for categorical variables. Comparisons between paired measurements obtained on ECPELLA support day 1 and the final day of V-A ECMO support were performed using the Wilcoxon signed-rank test.

The relationships between EtCO_2_ and PAPP were assessed using Spearman’s rank correlation coefficient. The 95% confidence intervals (CIs) for Spearman’s rank correlation coefficients were estimated using bias-corrected and accelerated bootstrap resampling with 10,000 resamples. To further investigate the physiological association between EtCO_2_ and PAPP, paired hemodynamic data collected at 24-h intervals during ECPELLA support were evaluated using a linear mixed-effects model with PAPP as the dependent variable, EtCO_2_ as a fixed effect, and patient-specific random intercepts to account for repeated measurements. An additional mixed-effects model including ECBF and Impella pump flow as fixed effects was performed to account for concurrent changes in ECPELLA support. Spearman’s rank correlation and repeated-measures correlation analyses were also performed to further characterize the association between EtCO_2_ and PAPP.

Supplementary analyses were performed to explore the influence of respiratory factors on EtCO_2_, including correlations with PaCO_2_ and expiratory minute ventilation (MVe). Since PaCO_2_ and EtCO_2_ measurements were not perfectly synchronized, the PaCO_2_–EtCO_2_ gradient was not formally evaluated.

All statistical analyses were conducted using Jamovi (version 2.6.17; The Jamovi Project, Sydney, Australia). A two-sided *p*-value < 0.05 was considered statistically significant.

## 3. Results

### 3.1. Patient Characteristics

During the study period, V-A ECMO was initiated in 65 patients, of whom 26 also received Impella support. EtCO_2_ measurements were available in 24 of 26 patients (92%), whereas PAPP was measured in 16 of 26 patients (62%) (Figure 1).

Baseline demographic and clinical characteristics are summarized in Table 1. No statistically significant differences were observed between patients with and without PAC placement, except for BSA. V-A ECMO preceded the establishment of ECPELLA in 25 of the 26 patients, including two patients in whom mechanical circulatory support was escalated from V-A ECMO with IABP to ECPELLA. In one patient, Impella support preceded V-A ECMO. Among the six patients who did not undergo V-A ECMO decannulation, all subsequently transitioned to palliative care and died without V-A ECMO decannulation. The transition to palliative care was related to refractory circulatory failure (n = 2), major bleeding (n = 1), multiorgan failure (n = 1), limitation of further treatment in accordance with the patient’s/family’s wishes (n = 1), or sepsis (n = 1). Detailed patient-level clinical characteristics, including ECPR status, arrest cause, initial rhythm, and major clinical diagnoses, are provided in Table S1.

Hemodynamic, laboratory, and treatment parameters on ECPELLA support day 1, on the final day of V-A ECMO support, and their changes during ECPELLA support are presented in Table 2.

From ECPELLA support day 1 to the final day of V-A ECMO support, lactate levels decreased and bicarbonate levels increased (Table 2). PAPP, Mean-PAP, Native-CO and EtCO_2_ also increased over the study period. ECMO blood flow decreased, whereas Impella pump flow increased.

The time interval between PaCO_2_ sampling and EtCO_2_ measurement was 43 (11–79) minutes on day 1 and 51 (16–92) minutes on the final day of V-A ECMO support. Echocardiographic measurements, particularly left ventricular ejection fraction (LVEF), and pulmonary capillary wedge pressure (PCWP) were not consistently available and were therefore excluded from the analysis.

### 3.2. Correlation Between EtCO_2_ and Pulmonary Artery Pulse Pressure

Spearman correlation analysis showed that PAPP was significantly correlated with EtCO_2_ on ECPELLA support day 1 (*r* = 0.628, 95% CI: 0.137 to 0.871, *p* = 0.009) and on the final day of V-A ECMO support (*r* = 0.667, 95% CI: 0.036 to 0.947, *p* = 0.007). Changes in PAPP from ECPELLA support day 1 to the final day of V-A ECMO support were also significantly correlated with changes in EtCO_2_ (*r* = 0.759, 95% CI: 0.442 to 0.926, *p* = 0.001) (Figure 2).

Supplementary correlation analyses showed a significant correlation between EtCO_2_ and PaCO_2_ on the final day of V-A ECMO support, whereas no significant correlations were observed at other time points or with minute ventilation (Table S2).

### 3.3. Correlation Between PAPP and EtCO_2_ over 24 Hours

A total of 57 paired PAPP and EtCO_2_ measurements were obtained from 16 patients at 24-h intervals. In the linear mixed-effects model accounting for patient-level repeated measurements, EtCO_2_ was positively associated with PAPP (coefficient = 0.311, 95% CI 0.236–0.387, *p* < 0.001). This association remained significant after additional adjustment for ECBF and Impella pump flow (coefficient = 0.219, 95% CI 0.119–0.319, *p* < 0.001) (Table S3). Repeated-measures correlation analysis also showed a positive within-patient association between PAPP and EtCO_2_ (*rrm* = 0.745, 95% CI: 0.570–0.855, *p* < 0.001). The unadjusted Spearman correlation across the 57 paired measurements was also positive (*r* = 0.760, *p* < 0.001) (Figure S1). EtCO_2_ was not significantly correlated with MVe (*r* = −0.153, *p* = 0.255) and showed a modest correlation with PaCO_2_ (*r* = 0.322, *p* = 0.014). The time interval between PaCO_2_ and EtCO_2_ measurements was 46 (19–71) minutes.

## 4. Discussion

A positive association between PAPP and EtCO_2_ was observed on ECPELLA support day 1, on the final day of V-A ECMO support, in their respective changes over time, and across paired measurements collected at 24-h intervals. Although the confidence intervals for the individual time-point correlations were wide, reflecting the limited sample size, the association across repeated measurements was maintained in a linear mixed-effects model accounting for patient-level clustering and remained positive after additional adjustment for ECBF and Impella pump flow. These findings suggest that EtCO_2_ may reflect trends in PAPP during ECPELLA support, although the magnitude of this association remains uncertain.

At the group level, from ECPELLA support day 1 to the final day of V-A ECMO support, lactate levels decreased and bicarbonate levels increased, while PAPP, Mean-PAP, Native-CO, and EtCO_2_ increased, accompanied by a decrease in ECBF and an increase in Impella pump flow. The relatively low ECBF and lactate levels on ECPELLA support day 1 should be interpreted in the context of the timing of data acquisition and the course of MCS before the establishment of ECPELLA, as measurements were obtained after Impella implantation and ICU admission rather than during the initial phase of circulatory support. At the individual level, however, changes in PAPP and EtCO_2_ from day 1 to the final day did not show a uniform increasing trend; some patients showed only small changes, whereas others showed decreases in both variables. Given that 6 of the 16 patients were not successfully decannulated from V-A ECMO, these differing temporal patterns may reflect diverse hemodynamic trajectories during ECPELLA support, including recovery or deterioration, rather than a uniform recovery process.

In contrast, pulmonary artery pulsatility index (PAPi), a commonly used hemodynamic marker of RV function [26,27], did not show a significant change in this study. Because PAPi is determined by the ratio of PAPP to RAP [24], concurrent changes in RAP, including those influenced by V-A ECMO support, may have attenuated the apparent changes in PAPi despite changes in PAPP and EtCO_2_. Figure S2 provides a supplementary conceptual illustration of these potential hemodynamic relationships during ECPELLA support.

Supplementary analyses were performed to assess potential respiratory influences on EtCO_2_. A positive correlation between EtCO_2_ and PaCO_2_ was observed on the final day of V-A ECMO support, whereas no clear associations with PaCO_2_ or minute ventilation were observed at the other assessed time points. Further studies in larger cohorts, allowing stratified assessment across different respiratory and hemodynamic conditions and levels of right ventricular function, are needed to better characterize the relationships among respiratory factors, EtCO_2_, and pulmonary artery hemodynamics during ECPELLA support.

Prior studies have shown that minimal EtCO_2_ signals can persist even under conditions of near-absent pulmonary arterial flow during CPB [21]. Consistent with this property, EtCO_2_ demonstrated a higher measurement availability than PAC parameters in the present study (92% vs. 62%). Unlike echocardiography or PAC-derived variables (Native-CO and PAP), EtCO_2_ does not require specialized imaging skills, catheter placement, or intermittent measurements. It can be continuously obtained, and its trends can be shared among the multidisciplinary ICU team, including clinical engineers and nurses. If future studies further clarify the relationships among temporal changes in EtCO_2_, PAPP, and pulmonary blood flow, EtCO_2_ may provide a noninvasive and continuous source of monitoring information during ECPELLA management.

### Limitations

This study has several limitations.

First, this was a retrospective, single-center observational study with a small sample size, which limits the generalizability of the findings and may introduce selection bias. In particular, PAC placement was performed at the treating physician’s discretion, and PAPP was available in only 16 of the 26 patients receiving ECPELLA support. Although no statistically significant differences were detected between patients with and without PAC placement except for BSA, selection bias related to PAC placement cannot be excluded.

Second, the analyses at 24-h intervals included repeated measurements from individual patients and were exploratory in nature. Although a linear mixed-effects model was used to account for within-patient clustering and yielded a similar association between PAPP and EtCO_2_, these findings should be interpreted cautiously given the limited number of patients.

Third, although the mixed-effects analysis adjusted for ECBF and Impella pump flow, residual confounding from concurrent hemodynamic changes during ECPELLA support cannot be excluded. Given that most patients underwent ECPR, diverse hemodynamic changes associated with post-cardiac arrest syndrome, including myocardial stunning, may have occurred during the observation period and affected preload, RV–PA coupling, and biventricular interaction. Therefore, the observed association between PAPP and EtCO_2_ may partly reflect these concurrent hemodynamic changes.

Fourth, EtCO_2_ is influenced by ventilatory and metabolic factors. Although ventilator settings were adjusted to maintain a stable PaCO_2_ range, PaCO_2_ and EtCO_2_ were not measured simultaneously as part of routine clinical care. The wide confidence intervals in the supplementary analyses and the time interval between PaCO_2_ and EtCO_2_ measurements limit the ability to exclude respiratory confounding, as respiratory and hemodynamic conditions may have changed during this interval.

## 5. Conclusions

The present findings suggest that EtCO_2_ may provide noninvasive information on changes in PAPP during ECPELLA support. However, given the small sample size, further studies in larger cohorts are needed to better characterize this relationship and determine the clinical utility of EtCO_2_ for monitoring PAPP.

## Figures and Tables

**Figure 1:**
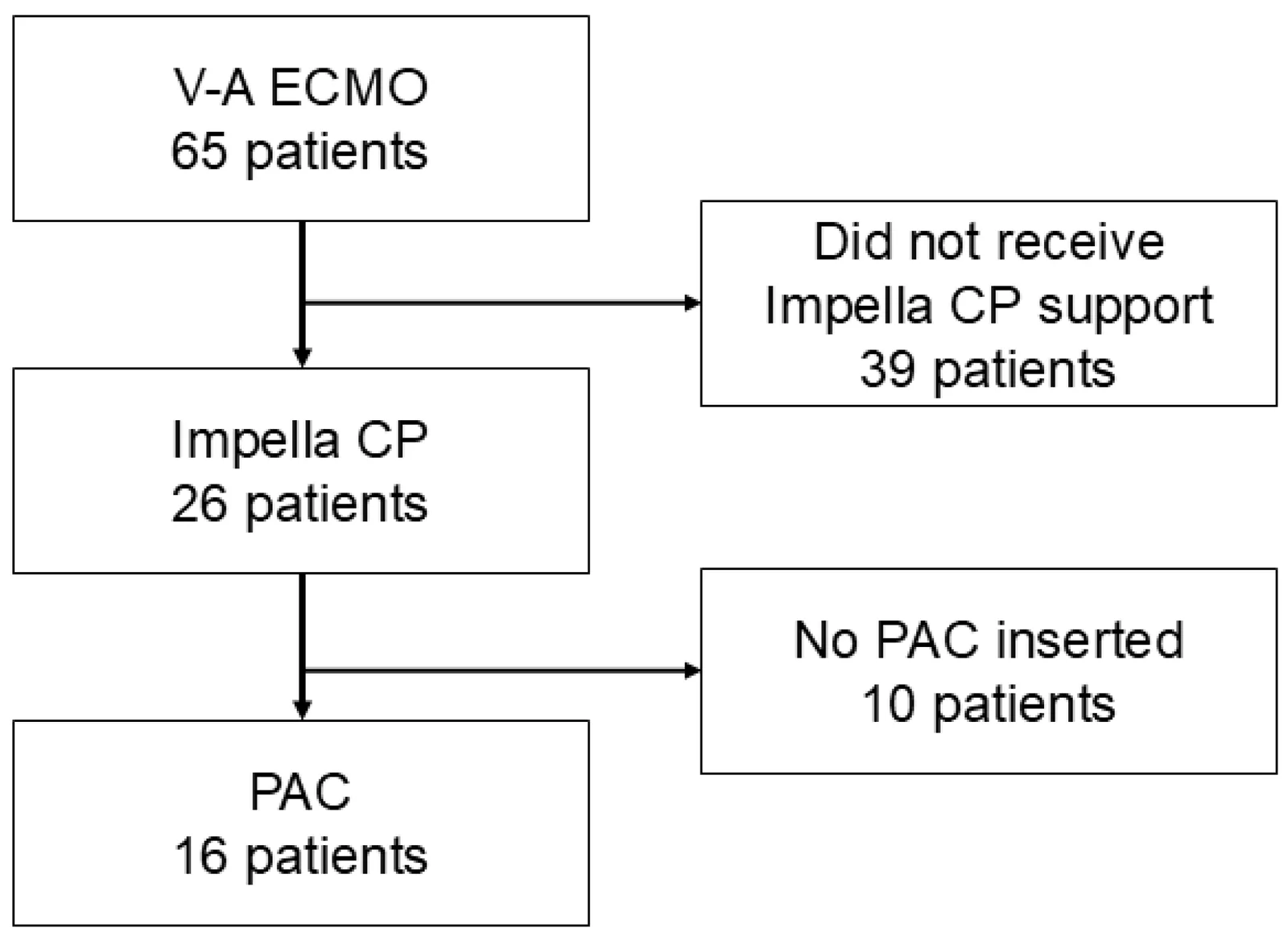
Patient selection flowchart. Among 65 patients receiving V-A ECMO, 26 were treated with combined V-A ECMO and Impella support. Pulmonary artery catheters were inserted in 16 patients at the discretion of the treating physicians. Due to the retrospective nature of the study, reasons for non-insertion were not consistently documented.

**Figure 2:**
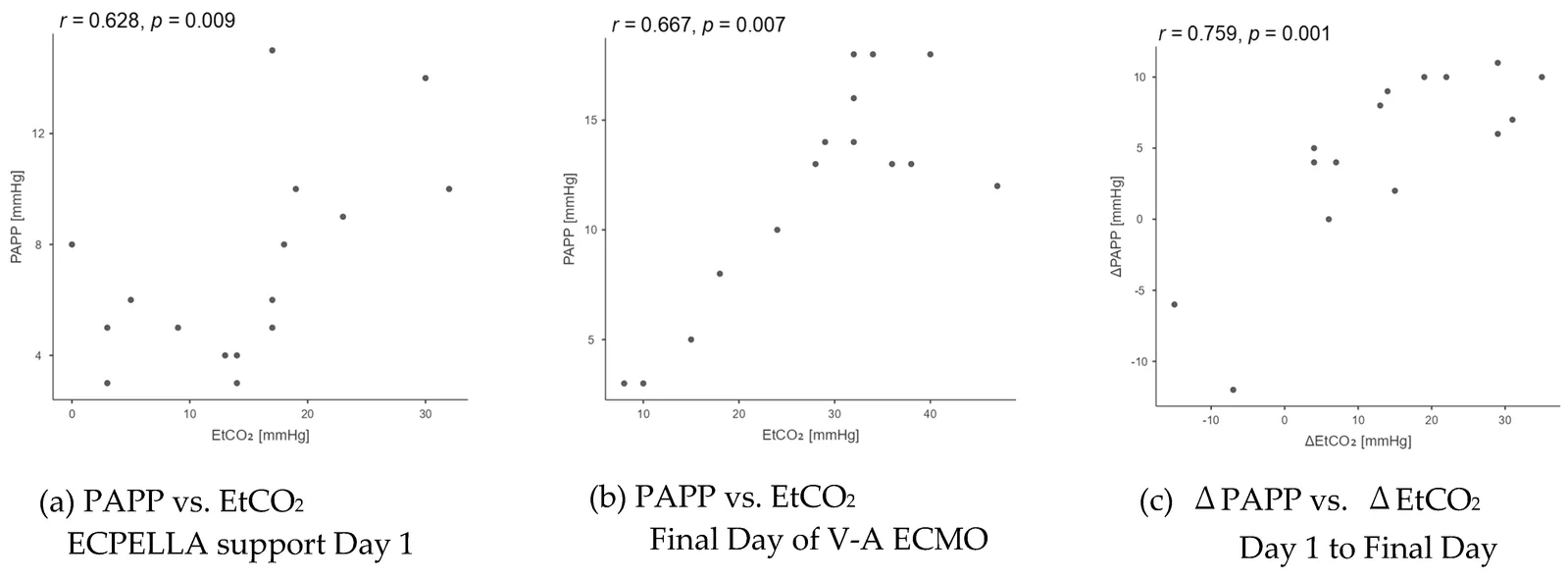
Correlation between end-tidal carbon dioxide (EtCO_2_) and pulmonary artery pulse pressure (PAPP). (**a**) EtCO_2_ versus PAPP on ECPELLA support day 1. (**b**) EtCO_2_ versus PAPP on the final day of V-A ECMO support. (**c**) Changes in PAPP (ΔPAPP) versus changes in EtCO_2_ (ΔEtCO_2_) from ECPELLA support day 1 to the final day of V-A ECMO support. Spearman’s rank correlation coefficients and corresponding *p*-values are shown in each panel.

**Table 1:** Clinical characteristics and outcomes of patients with and without pulmonary artery catheterization.

Demographics	No PAC Inserted [n = 10]	PAC Inserted [n = 16]	*p*-Value
Age [years]	70.5 (62.3–76)	71.5 (66.0–77.0)	0.427
Male sex	8, (80.0)	14, (87.5)	0.625
Body surface area [m^2^]	1.57 (1.53–1.70) [n = 7]	1.81 (1.76–1.91)	0.004
**E** **CMO** **indication**			0.617
**Extracorporeal cardiopulmonary resuscitation**	9, (90.0)	12, (75.0)	
Myocardial infarction	8, (88.9)	9, (75.0)	
Mechanical complications of myocardial infarction	0, (0.0)	1, (8.3)	
Heart failure	0, (0.0)	1, (8.3)	
Arrhythmia	0, (0.0)	1, (8.3)	
Hypothermia	1, (11.1)	0, (0.0)	
**Cardiogenic shock**	1, (10.0)	4, (25.0)	
Myocardial infarction	1, (100.0)	1, (25.0)	
Mechanical complications of myocardial infarction	0, (0.0)	1, (25.0)	
Postoperative low cardiac output syndrome	0, (0.0)	1, (25.0)	
Other	0, (0.0)	1, (25.0)	
**MCS sequence before ECPELLA**			0.692
V-A ECMO	10, (100.0)	13, (81.3)	
V-A ECMO with IABP	0, (0.0)	2, (12.5)	
Impella	0, (0.0)	1, (6.3)	
**Device-related complications**			
Major bleeding	3, (30.0)	4, (25.0)	1.000
Access-related bleeding	6, (60.0)	8, (50.0)	0.701
Limb ischemia	0, (0.0)	2, (12.5)	0.508
Hemolysis	1, (10.0)	2, (12.5)	1.000
Thrombosis	0, (0.0)	0, (0.0)	-
Device displacement	0, (0.0)	1, (6.3)	1.000
Neurological complications	2, (20.0)	4, (25.0)	1.000
**S** **upport duration**			
Duration of ECMO support [hours]	74.05 (32.25–88.43)	81.79 (63.24–112.24)	0.317
Duration of Impella support [hours]	93.85 (37.35–118.70)	121.05 (66.00–169.05)	0.155
ECMO decannulation	4, (40.0)	10, (62.5)	0.422
Survival to discharge	4, (40.0)	4, (25.0)	0.665

Values are n (%) or median (interquartile range). The number of patients analyzed for each variable may vary because of missing data. For subcategories of ECMO indication, percentages were calculated using the number of patients within the respective indication (ECPR or cardiogenic shock) as the denominator. Continuous variables were compared using the Mann–Whitney U test, and categorical variables were compared using Fisher’s exact test or the Fisher–Freeman–Halton exact test, as appropriate.

**Table 2:** Hemodynamic, laboratory, and treatment parameters on ECPELLA support day 1, on the final day of V-A ECMO support, and their changes during ECPELLA support.

	ECPELLA Support Day 1	Final Day of V-A ECMO	Day 1 to Final Day	*p*-Value
**Laboratory**				
Lactate [mmol/L]	2.3 (1.4–4.2)	1.2 (0.9–1.4)	−0.9 (−3.9–0.7)	0.001
PaCO_2_ [mmHg]	33.9 (30.4–38.8)	36.5 (34.9–39.5)	2.4 (−0.9–6.7)	0.135
HCO_3_^−^ [mmol/L]	21.2 (20.6–23.3)	25.2 (24.1–28.0)	4.3 (2.5–7.3)	0.002
pH	7.41 (7.38–7.47)	7.43 (7.41–7.45)	0.01 (−0.06–0.08)	0.720
**Hemodynamics**				
RAP [mmHg]	14 (8–16)	16 (11–16)	3 (−3–6)	0.064
Systolic-PAP [mmHg]	23 (21–27)	31 (23–36)	8 (−4–13)	0.064
Diastolic-PAP [mmHg]	16 (13–20)	17 (15–21)	0 (−2–6)	0.356
Mean-PAP [mmHg]	18 (16–22)	22 (18–26)	3 (0–6)	0.048
Systolic-ABP [mmHg]	84 (78–91)	100 (83–120)	9 (−11–29)	0.193
Diastolic-ABP [mmHg]	75 (64–85)	68 (62–78)	−14 (−26–11)	0.083
Mean-ABP [mmHg]	76 (72–89)	80 (67–87)	−9 (−19–18)	0.421
PAPP [mmHg]	6 (5–9)	13 (9–15)	6 (2–10)	0.041
PAPi	0.54 (0.31–1.38)	0.81 (0.53–1.25)	0.15 (−0.30–0.35)	0.847
Native-CO [L/min]	1.7 (1.4–1.9)	3.3 (2.5–5.4)	0.5 (0.0–3.0)	0.031
EtCO_2_ [mmHg]	16 (8–18)	32 (21–35)	14 (5–27)	0.004
**Treatment**				
ECMO blood flow [L/min]	2.6 (2.1–2.9)	1.7 (1.4–2.0)	−0.9 (−1.2–−0.5)	0.009
Sweep gas flow [L/min]	2.0 (1.5–2.0)	1.5 (1.5–1.8)	0.0 (0.0–0.5)	0.097
Impella pump flow [L/min]	1.8 (1.0–2.2)	2.7 (1.7–3.2)	0.9 (0.5–1.5)	0.001
PEEP [cmH_2_O]	10 (10–11)	10 (10–10)	0 (0–1)	0.563
Expiratory minute ventilation [L/min]	5.0 (4.5–6.3)	5.8 (4.2–6.4)	0.2 (−0.7–1.6)	0.903
Dobutamine dose [μg/kg/min]	0.0 (0.0–1.0)	0.0 (0.0–2.8)	0.0 (0.0–2.0)	0.547

Values are presented as median (interquartile range). *P*-values were calculated using the Wilcoxon signed-rank test for paired comparisons between ECPELLA support day 1 and the final day of V-A ECMO support. **Abbreviations:** ABP, arterial blood pressure; PEEP, positive end-expiratory pressure.

## Data Availability

The datasets generated and/or analyzed during the current study are available from the corresponding author upon reasonable request due to patient privacy restrictions.

## Supplementary Materials

The following supporting information can be downloaded at https://www.mdpi.com/article/10.3390/jcm15208070/s1. Table S1. Patient characteristics. Table S2. Correlation between end-tidal carbon dioxide (EtCO_2_) and respiratory parameters during ECPELLA support. Table S3. Linear mixed-effects analysis of the association between EtCO_2_ and PAPP accounting for repeated measurements. Figure S1: Correlation between pulmonary artery pulse pressure (PAPP) and end-tidal carbon dioxide (EtCO_2_) measured every 24 h in 16 patients. Figure S2: Conceptual illustration of potential hemodynamic states during ECPELLA support.

## Abbreviations

The following abbreviations are used in this manuscript:

PAPP	pulmonary artery pulse pressure
PAPi	pulmonary artery pulsatility index
EtCO_2_	end-tidal carbon dioxide
RAP	right atrial pressure (if RAP was not available, central venous pressure [CVP] was used as a substitute)
ECPELLA	V-A ECMO-assisted Impella support
PAC	pulmonary artery catheter
MVe	expiratory minute ventilation
PaCO_2_	arterial carbon dioxide tension
