# Supplementary material for: Association Between End-Tidal Carbon Dioxide and Pulmonary Artery Pulse Pressure During Veno-Arterial Extracorporeal Membrane Oxygenation with Impella®: A Retrospective Exploratory Study
Source: J Clin Med. 2026 Oct 17;15(20):8070. doi: 10.3390/jcm15208070 (PMC13706618; doi:10.3390/jcm15208070)
Supplement: Supplementary file 1 [file jcm-15-08070-s001.zip › jcm-4587328-supplementary.pdf]

Table S1. Patient characteristics.

No	Primary diagnosis	ECPR	Arrest cause	Initial rhythm	Mechanical complication	Clinical detail
1	STEMI	Yes	AMI	VF	-	
2	NSTEMI	Yes	AMI	pVT	-	
3	STEMI	Yes	AMI	PEA	-	
4	STEMI	Yes	Arrhythmia	VF	-	PVC-triggered VF storm
5	STEMI	No	-	-	-	
6	Heart failure	Yes	Arrhythmia	VF	-	Heart failure due to ischemic heart disease
7	STEMI	Yes	AMI	VF	-	
8	STEMI	Yes	AMI	VF	-	
9	VSP	Yes	Mechanical complication	PEA	VSP	
10	STEMI	Yes	AMI	PEA	-	
11	VSP	No	-	-	VSP	
12	STEMI	No	-	-	-	Impella-related event requiring V-A ECMO
13	STEMI	Yes	AMI	pVT	-	
14	NSTEMI	Yes	AMI	Asystole	-	
15	STEMI	No	-	-	PMR	Postoperative LCOS after MVR and CABG
16	STEMI	Yes	AMI	VF	-	

Abbreviations: ECPR, extracorporeal cardiopulmonary resuscitation; STEMI, ST-elevation myocardial infarction; NSTEMI, non-ST-elevation myocardial infarction; AMI, acute myocardial infarction; PVC, premature ventricular contraction; VF, ventricular fibrillation; pVT, pulseless ventricular tachycardia; PEA, pulseless electrical activity; VSP, ventricular septal perforation; PMR, papillary muscle rupture; LCOS, low cardiac output syndrome; MVR, mitral valve replacement; CABG, coronary artery bypass grafting.

Table S2. Correlation between end-tidal carbon dioxide ($EtCO_2$) and respiratory parameters during ECPELLA support.

	**Coefficients (*r*)**	**95% CI**	***p*-value**
**$EtCO_2$ [mmHg] vs $PaCO_2$ [mmHg]**			
ECPELLA support day 1	-0.065	-0.587 to 0.570	0.811
Final day of V-A ECMO	0.579	0.077 to 0.896	0.024
Day 1 to final day	-0.014	-0.594 to 0.628	0.959
**$EtCO_2$ [mmHg] vs MVe [L/min]**			
ECPELLA support day 1	-0.084	-0.584 to 0.473	0.756
Final day of V-A ECMO	-0.086	-0.638 to 0.455	0.761
Day 1 to final day	-0.490	-0.874 to 0.248	0.064

Spearman's rank correlation coefficients are presented with 95% confidence intervals estimated using bias-corrected and accelerated bootstrap resampling with 10,000 resamples. CI, confidence interval; $EtCO_2$, end-tidal carbon dioxide; $PaCO_2$, arterial partial pressure of carbon dioxide; MVe, minute ventilation.

Table S3. Linear mixed-effects analysis of the association between $EtCO_2$ and PAPP accounting for repeated measurements.

**Model**	**Fixed effect**	**Estimate**	**95% CI**	***p*-value**
Linear regression analysis	$EtCO_2$	0.315	0.239 to 0.392	<0.001
Linear mixed-effects model patient-level random intercepts	$EtCO_2$	0.311	0.236 to 0.387	<0.001
Linear mixed-effects model adjusted for ECBF and Impella pump flow	$EtCO_2$	0.219	0.119 to 0.319	<0.001
	ECBF	-0.844	-2.471 to 0.783	0.303
	Impella pump flow	1.337	-0.091 to 2.766	0.067

The linear mixed-effects models included PAPP as the dependent variable, $EtCO_2$ as a fixed effect, and patient-specific random intercepts to account for repeated measurements within individual patients. The adjusted model additionally included ECMO blood flow (ECBF) and Impella pump flow as fixed effects. Estimates represent the fixed-effect regression coefficients. The analyses included 57 paired measurements from 16 patients. CI, confidence interval; ECBF, ECMO blood flow; $EtCO_2$, end-tidal carbon dioxide; PAPP, pulmonary artery pulse pressure.

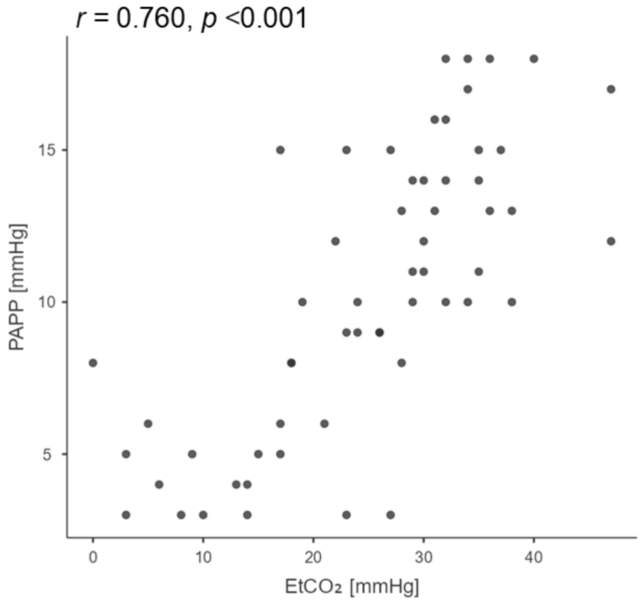

Figure S1. Correlation between pulmonary artery pulse pressure (PAPP) and end-tidal carbon dioxide ($EtCO_2$) measured every 24 hours in 16 patients. A total of 57 paired PAPP and $EtCO_2$ measurements were obtained from 16 patients at 24-hour intervals. Unadjusted Spearman correlation analysis showed a significant positive correlation between PAPP and $EtCO_2$ ($r = 0.760$, $p < 0.001$). Each dot represents a single measurement.

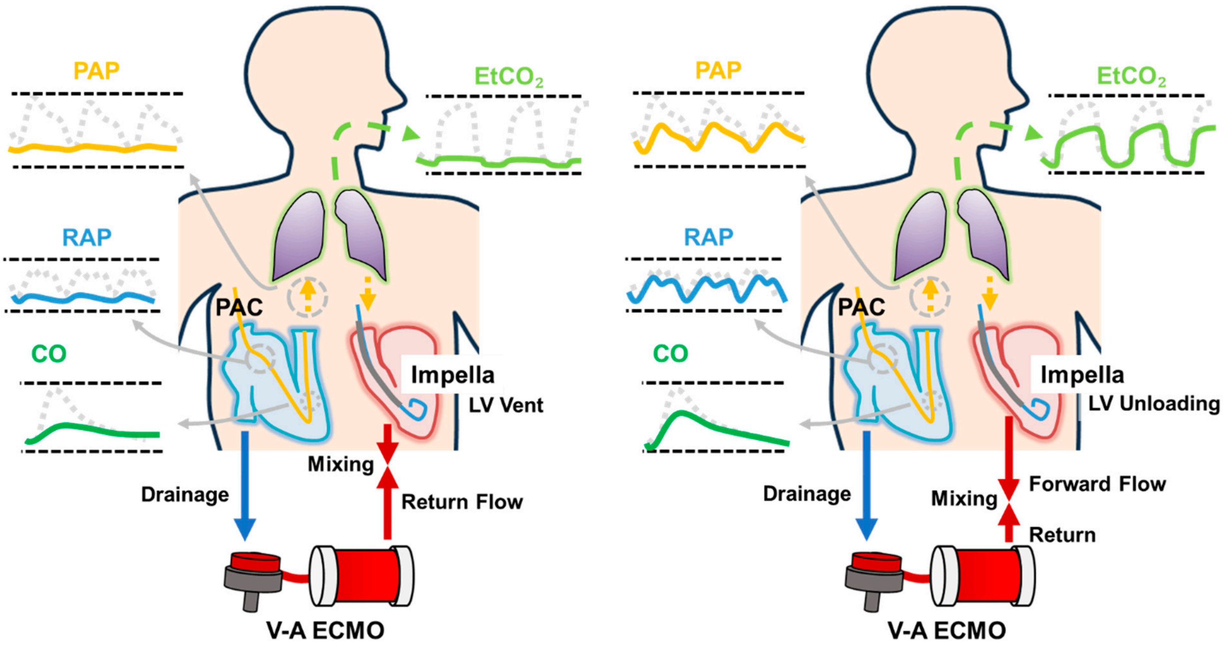

(a) Hemodynamic state A **(b) Hemodynamic state B**

Figure S2. Conceptual illustration of potential hemodynamic states during ECPELLA support. These scenarios are not models validated in the present study but are intended to facilitate interpretation of potential hemodynamic differences. (a) A hemodynamic state with a relatively greater contribution of V-A ECMO support and (b) a hemodynamic state with relatively greater contributions of native pulmonary circulation and Impella support are conceptually illustrated. Differences in the relative contributions of V-A ECMO, native pulmonary circulation, and Impella support may result in different hemodynamic profiles of RAP, PAP, CO, and $EtCO_2$. Because PAPi is calculated as PAPP divided by RAP, PAPP and PAPi may not necessarily change in parallel across these different hemodynamic states.
